# T253. THE CORRELATION ANALYSIS BETWEEN RENAMING SCHIZOPHRENIA AND VISITING FREQUENCY OF MENTAL HEALTH SERVICES BY BIG DATA ANALYSIS (INTERNET SEARCHES AND NEWSPAPER ARTICLES) IN SOUTH KOREA

**DOI:** 10.1093/schbul/sby016.529

**Published:** 2018-04-01

**Authors:** Sang Yup Lee, Kyung Sue Hong, Yeon Ho Joo, Shinsuke Koike, Yu Sang Lee, Jun Soo Kwon

**Affiliations:** 1 Yonsei University; 2 Sungkyunkwan University School of Medicine, Samsung Medical Center; 3 University of Ulsan College of Medicine; 4 The University of Tokyo; 5 Yongin Mental Hospital; 6 Seoul National University College of Medicine

## Abstract

**Background:**

Korean Neuropsychiatric Association changed the Korean term for schizophrenia from ‘split-mind disorder’ to ‘attunement disorder’ in 2012, to dispel the stigma associated with name, and to promote early detection and treatment. Information on the internet affects the public awareness and attitude toward schizophrenia. The main purpose of this study was to investigate the correlation between renaming schizophrenia and the pattern of mental health services utilization by big data analysis of internet (newspaper articles and internet searches) in Korea.

**Methods:**

From January 2016 to September 2017, newspaper articles on “attunement disorder” and “split-mind disorder” available on the internet were classified as related with negative images like crime and helpful or positive in dispelling the stigma. The relationship between the number of anti-stigma newspaper articles and newspaper articles of schizophrenia containing both positive and negative images was examined. In addition, using Naver, a major internet search engine in Korea, we investigated the total number of internet searches of both old and new name of schizophrenia by gender differences. Finally, the frequency of the visits of mental health services of patients with schizophrenia was measured using the Korean Healthcare Bigdata Hub (http://opendata.hira.or.kr/home.do#none) for 14 months and the correlation between the frequency of the visits and the above big data was examined. The data were analyzed using the SPSS/WIN 24.0. Pearson correlation coefficients were used to analyze correlations.

**Results:**

The amounts of newspaper articles containing anti-stigma of schizophrenia were correlated with the amounts of newspaper articles containing negative images like crime of the new name (attunement disorder) of schizophrenia (r=0.528, p<0.01), which was greater than the amounts of newspaper articles containing the old name (split-mind disorder) of schizophrenia (r=0.300, p<0.01). We also found that a strong positive correlation between the number of articles about “attunement disorder” and search frequency about the term on the internet. In addition, the search frequency was more highly related to the number of articles containing negative images of the illness (e.g., related crimes, r = 0.910, p<0.01) than that of articles providing positive aspects of the illness (e.g., dispelling stigma, r = 0.423, p<0.01). There was no significant correlation between the number of schizophrenia-related newspaper articles in previous month and the visits of mental health services of patients with schizophrenia in next month. There were no gender differences in internet searches. The correlation between the internet search frequency for “attunement disorder” in the previous month and the visits of the mental health services of patients with schizophrenia (r = 0.185, p>0.05) in next month was larger than the correlation of “split-mind disorder” searches with mental health services utilization (r = 0.082, p>0.05).

**Discussion:**

“Attunement disorder” rather than “split-mind disorder” was appeared more frequently in newspaper articles of the anti-stigma characteristics. “Attunement disorder” seems to be more useful for anti-stigma campaign. Renaming schizophrenia didn’t seem to affect the visiting frequency of mental health services. There was statistical limitation which was originated from the lack of numbers of patient’s information. It was because Korean Bigdata Hub provided patients information just for 14 months as monthly data. Also, it should be considered that the time period, the kinds of mental disorders and the search engine we investigated were limited. Future research needs to overcome these limitations.

